# Elicitor‐mediated simultaneous accumulation of phloridzin and ursolic acid in Annurca apple peel‐derived calli

**DOI:** 10.1002/jsfa.13955

**Published:** 2024-10-10

**Authors:** Carmen Laezza, Maria Maisto, Paola Imbimbo, Daria Maria Monti, Mariavittoria Verrillo, Antonio Di Loria, Simona Maria Monti, Adua Marzocchi, Paolo Grieco, Gian Carlo Tenore, Vincenzo D'Amelia, Maria Manuela Rigano

**Affiliations:** ^1^ Department of Agricultural Sciences University of Naples Federico II Naples Italy; ^2^ Immunoveg s.r.l. c/o Naples Italy; ^3^ ChimNutra Labs, Department of Pharmacy University of Naples Federico II Naples Italy; ^4^ Department of Chemical Sciences University of Naples Federico II Naples Italy; ^5^ Department of Veterinary Medicine and Animal Production University of Naples Federico II Naples Italy; ^6^ Institute of Biostructures and Bioimaging, National Research Council Naples Italy; ^7^ Department of Pharmacy University of Naples Federico II Naples Italy

**Keywords:** Rosaceae, callus cultures, dihydrochalcone, terpene, elicitor, foodborne pathogens

## Abstract

**BACKGROUND:**

Apple peel is rich in natural molecules, many exhibiting a significant bioactivity. In this study, our objective was to establish a novel callus line derived from the apple peel of the Italian local variety Annurca, known to accumulate high levels of dihydrochalcones and terpenes. In this regard, we tested the impact of one elicitor, yeast extract, on the expression of genes encoding key enzymes involved in phloridzin and ursolic acid biosynthesis, leading to the accumulation of these antioxidant compounds. We also assessed the bioactivity of callus extracts enriched in these phytochemicals.

**RESULTS:**

After the elicitation, data showed increased expression of genes directly related to the synthesis of phloridzin and ursolic acid that were found to accumulate within the cultures. This presumably could explain the remarkable activity of extracts from the elicited‐calli in inhibiting the growth of *Staphylococcus aureus* and *Bacillus cereus*. Also, the extracts enriched in antioxidant compounds inhibited reactive oxygen species (ROS) production in human cells exposed to ultraviolet‐A (UV‐A) radiation.

**CONCLUSION:**

Our results underscore the vast potential of the Annurca apple peel cell line in producing natural compounds that can be employed as food components to promote human health. © 2024 The Author(s). *Journal of the Science of Food and Agriculture* published by John Wiley & Sons Ltd on behalf of Society of Chemical Industry.

## INTRODUCTION

Plants produce a broad array of specialized metabolites of commercial interest, thanks to their extensive metabolic enzymatic repertoire.^1^ For instance, polyphenols may serve as food supplements with anti‐inflammatory, anticancer, and antihypertensive properties,^2^ and terpenes may be used as natural fragrances or food additives with wide beneficial effects.^3^

Different strategies have been proposed to maximize the production of these molecules in plants, such as the plant cell culture (PCC). These are ‘natural biochemical machines’, cultivated in a medium consisting of few and simple ingredients (i.e., salts, sugar, vitamins, and phytohormones), thus overcoming the limit of the seasonality.^4^ This ready‐to‐use technology has already been adopted by cosmetic and food industries, as demonstrated by the food additives TEUPOL 50P, ECHINAN 4P and ACTEOS 10P (ABResearch srl, Brendola, Italy) produced by cell cultures from *Ajuga reptans*, *Echinacea angustifolia* and *Aloysia citrodora*, respectively. That said, the selection of plant species to develop PCCs is crucial and is based on its metabolic profile.

Taking into account the earlier considerations, we previously devised a novel protocol for the production and elicitation of PCCs derived from the pulp of the Annurca apple, a *Malus pumila* ecotype native to southern Italy and recognized by European Council as Protected Geographical Indication (PGI).^5^ This ecotype was chosen since extracts obtained from the apple tissues demonstrated their potential in the treatment of diabetes^6^ as well as skin ageing.^7^ Indeed, Annurca apple contains high concentrations of both polyphenols [114.99 mg 100 g^−1^ fresh weight (FW)] and triterpenoids (784.40 μg mL^−1^), with phloridzin and ursolic acid contributing significantly to the antioxidant activity of its extracts.^7^ However, the biochemical composition of the pulp and the peel of this apple can largely vary and therefore the bioactivity of their extracts.^8^ In this study, we used the peel of Annurca apple as a source for cell cultivation. This tissue, which is the outermost layer of the fruit, plays a crucial role against pathogens, accumulating a diverse range of potentially bioactive metabolites. We also aimed to explore if the unique properties of the apple peel may have a positive impact on the bioactive potential of the peel‐derived cell cultures. Once these callus cultures were established, yeast extract (YE) was used as biotic elicitor to further stimulate the accumulation of bioactive compounds. The effect of YE on Annurca cell cultures was investigated by targeted metabolomic along with transcriptomic analyses to identify both the metabolites and the YE‐responsive genes in elicited callus cultures. Extracts from the apple peel are also known to possess antioxidant and antibacterial activities.^9^ These extracts proved to extend food shelf‐life, preserving it against foodborne pathogens (i.e., *Escherichia coli*, *Bacillus cereus* and *Staphylococcus aureus*)^10^ and to significantly increase cell viability and decrease cell death in ultraviolet (UV) exposed cells.^11^ Based on these studies, here we also investigated the antioxidant and antibacterial effect of Annurca PCC extracts to elucidate the possible correlation between the callus culture metabolic reservoir and the biological activities.

## MATERIALS AND METHODS

### Chemicals and reagents

All chemicals, reagents, and standards used were either analytical, LC–MS or high‐performance liquid chromatography (HPLC) grade reagents. Milli‐Q water (Millipore, Bedford, MA, USA) was used. Gallic acid (purity ≥ 98% HPLC), procyanidin B1 (purity ≥ 90% HPLC), procyanidin B2 (purity ≥ 90% HPLC), (−)‐catechin (purity ≥ 98% HPLC), (−)‐epicatechin (purity ≥ 98% HPLC), chlorogenic acid (purity ≥ 95% HPLC), rutin (purity ≥ 94% HPLC), ursolic acid (purity ≥ 98.5% HPLC) quercetin 3‐*O*‐glucoside (purity ≥ 98% HPLC), phloridzin (purity ≥ 99% HPLC) were from Sigma‐Aldrich (Milan, Italy). Chemicals for Gamborg B5 medium, plant growth regulators and sucrose were from Duchefa Biochemie (RV Haarlem, The Netherlands). Yeast extract was from Thermo Fisher Scientific (Waltham, MA, USA).

### Callus culture establishment, YE treatment and callus biomass measurement

Annurca apple fruits were collected in Valle di Maddaloni (Caserta, Italy). The sterilization and the production of peel‐derived calli were performed as described in the literature.^5^ Calli from exponential developmental stage (after 10 days of growth) were incubated in the cultivation medium supplemented with either 300 or 500 mg L^−1^ YE (referred to as YE 300 and YE 500, respectively) in the dark at 25 ± 2 °C, for 3 weeks. At the end of the treatment, calli were ground in liquid nitrogen with a mortar and pestle and the powder was stored at −80 °C before further analyses. After YE treatment, the increase in callus biomass was measured as dry weight (DW). Calli were taken out from culture Petri dishes, washed with sterile water, and dried at 60 °C for 48 h to determine the DW.

### Extract preparation

*Extraction A*. Quantitative polyphenol content was determined according to a method previously described with slight modifications.^12^ The extraction was performed adding 1 mL of 80% methanol containing 1% formic acid solution to 40 mg of lyophilized samples. Samples were first placed in an ultrasonic bath (Branson Fisher Scientific 150 E Sonic Dismembrator, Fisher Scientific International, Inc, Pittsburg, PA) for 10 min, then on an orbital shaker (Sko‐DXL; Argolab, Carpi, Italy) for 10 min, and centrifuged at 765 *g* for 10 min. The obtained pellets were re‐extracted with the same procedure using 0.5 mL of the same extraction solvents.

*Extraction B*. For the analysis of callus culture extract bioactivity, polyphenolic compounds were extracted in ethanol, a GRAS (generally recognized as safe) solvent, as previously reported,^13^ with some modifications. A volume of 12.5 mL of ethanol was added to 0.5 g of lyophilized biomass, and extractions were carried out in an ultrasonic bath for 1 h. Then, the samples were centrifuged at 5000 × *g*, supernatants collected and dried under nitrogen flux. The extracts were stored at −80 °C.

### Total polyphenol content and ABTS assays

Total phenolic content (TPC) was determined according to Singleton and Rossi^14^ with slight modifications. A mixture containing 62.5 μL of extract, 62.5 μL of Folin–Ciocalteu reagent and 250 μL of Milli‐Q water was incubated for 6 min. Then, 625 μL of 7.5% sodium carbonate and 500 μL of Milli‐Q water were added. After incubation for 90 min, absorbance was measured at 765 nm. The TPC was expressed as gallic acid equivalents (g kg^−1^ DW). To investigate the antioxidant activity, the ABTS (2,2'‐azino‐bis(3‐ethylbenzothiazoline‐6‐sulfonic acid)) analysis was performed as described by Ali *et al*.^15^ Briefly, 1 mL of ABTS radical cation was added to 100 μL of polyphenolic extract. The absorbance was measured at 734 nm after 2.5 min. The ABTS radical cation scavenging capacity of the extract was calculated as described in the literature.^15^

### HPLC‐DAD‐FLD polyphenol quantification

The determination of metabolites was performed on a Jasco Extrema LC‐4000 system (Jasco Inc., Easton, MD, USA) provided with photodiode array detector (DAD) and a fluorescence detector (FLD), coupled with an autosampler and a binary solvent pump. The column used was a Kinetex®C18 column (250 mm × 4.6 mm, 5 μm; Phenomenex, Torrance, CA, USA). The analyses were performed at a flow rate of 1 mL min^−1^, with solvent A (2% formic acid) and solvent B (0.5% formic acid in acetonitrile and water 50:50, *v/v*). The elution was performed according to a chromatographic method previously reported.^7^, ^8^ The quantitative determinations were performed using a calibration curves, elaborated at six different concentration levels, over the standard concentrations range of 0.1–0.0001 mg mL^−1^.

### Ursolic acid extraction and quantitative analysis by HPLC‐DAD

A volume of 5 mL of ethyl acetate was added to 100 mg of lyophilized samples. The mixture was placed in an ultrasonic bath (Branson Fisher Scientific 150 E Sonic Dismembrator) for 15 min and shaken on an orbital shaker (Sko‐DXL, Argolab) for 10 min. Then, samples were centrifuged at 25 000 × *g* for 10 min and the supernatants were collected and stored at 4 °C. The obtained pellets were re‐extracted with 5 mL of ethyl acetate using the same procedure. Finally, the extracts obtained were evaporated, reconstituted at 5 mg mL^−1^ in dimethylsulfoxide/acetonitrile, and stored at −20 °C. The analyses were carried out on a Jasco Extrema LC‐4000 HPLC system (Jasco Inc.), equipped with an autosampler, a binary solvent pump, and a DAD. Separation was performed according to the previously described and validated method.^7^ The quantification of ursolic acid was performed at 205 nm. Moreover, the sensitivity of the current method was described by an ursolic acid limit of detection (LOD) and limit of quantification (LOQ) of 0.298 and 0.845 ppm, respectively.

### RNA extraction and qRT‐PCR

The expression level of the genes 2‐alkenal reductase (NADP(^+^)‐dependent)‐like (*MdDH*), UDP‐glucose: phloretin 2′‐*O*‐glucosyltransferase (*MdUGT88F1*), oxydosqualene cyclase 1 (*MdOSC1*) and triterpene monooxygenase (*MdCYP716A175*) was analysed by quantitative reverse transcription polymerase chain reaction (qRT‐PCR). The RNA was extracted using ISOLATE II RNA Mini Kit (Bioline – Meridian Bioscience, London, UK) and converted into complementary DNA (cDNA) using Tetro cDNA Synthesis Kit (Bioline). For the qRT‐PCR reactions the SensiFAST SYBR Hi‐ROX Kit was used (Bioline). The primers used are listed in Supporting Information Table S1. The amplification was carried out using the 7900HT Fast‐Real Time PCR System (Applied Biosystems, Foster City, CA, USA), according to the following steps: 2 min at 50 °C, 2 min at 95 °C, 0.15 min at 95 °C and 50 °C for 1 min for 40 cycles. The amplification programme was followed by the thermal denaturing step (0.15 min at 95 °C, 0.15 min at 60 °C, 0.15 min at 95 °C) to generate the dissociation curves. All reactions were run in triplicate for each sample and housekeeping genes coding for the elongation factor 1‐alpha (Ef 1‐α) and actin (ACT) were used as reference genes. The expression levels relative to the reference genes were calculated using the formula 2−ΔCT, where ΔCT = (CT RNAtarget – CT reference RNA). The comparison of RNA expression was based on a comparative CT method (ΔCT) and the relative expression was quantified and expressed according to log_2_RQ, where RQ was calculated as 2−ΔΔCT and where ΔCT = (CT RNAtarget – CT reference RNA) – (CT calibrator – CT reference RNA).^16^ Non‐elicited calli were selected as calibrator.

GenBank accession numbers are as follows: EF 1‐ α, DQ341381.1; ACT, AB638619.1; DH, NC_041790.1; UGT88F1, NM_001328723.2; OSC1, NM_001294017.1; CYP716A175, EB148173.

### Disk diffusion method to test bacterial growth

The antibacterial test was conducted by diffusion disk methods in accordance with the standard method of the National Committee for Clinical Laboratory Standards (NCCLS).^17^ Bacterial strains selected for this analysis are *Staphylococcus aureus*, *Enterococcus faecalis*, *Bacillus cereus*, *Salmonella enterica*, *Escherichia coli* and *Listeria monocytogenes* provided by CERMANU (Interdepartmental Research Centre on Nuclear Magnetic Resonance for the Environment, Agro‐food and New Materials, University of Naples Federico II). When bacteria grown in solid medium are sensitive to the extract tested, microbial growth is reduced, and an inhibition zone becomes visible in the plate. Microbial cultures were transferred to nutrient agar and incubated at 37 °C for 24 h. The inoculum was standardized transferring colonies from the nutrient agar to sterile saline solution up to 108 CFU mL^−1^ (0.5 McFarland). Then, 200 μL of each culture were placed onto the surface of Mueller–Hinton agar, where several disks (6.0 mm diameter) were incubated with 20 μL of each methanolic extracts and placed at 37 °C for 24 h. Given the results from the metabolic analyses, the antimicrobial effect of control and YE 500‐elicited extracts was assessed. Sterile distilled water and a combination of ampicillin and clavulanic acid were employed as negative and positive control, respectively. Each experiment was performed in triplicate and the inhibition zones were calculated considering the total diameters in each plate.

The fold change (FC) was calculated using the following formula:

$$\mathrm{FC}=\frac{\text{diameter of inhibition of bacteria grown in the presence of the treated callus extract}}{\text{diameter of inhibition of bacteria grown in the presence of the control callus extract}}$$

### Cell culture and MTT and DCFDA assays

Immortalized human keratinocytes (HaCaT) were from Innoprot (Biscay, Spain). Cells were cultured in Dulbecco's modified Eagle's medium (DMEM), supplemented with 10% foetal bovine serum (HyClone), 2 mmol L^−1^ l‐glutamine and antibiotics, under a 5% carbon dioxide (CO_2_) humidified atmosphere at 37 °C. HaCaT cells were seeded in 96‐well plates at a cell density of 2 × 10^3^ cells well^−1^. After 24 h cells were incubated with increasing concentration (from 1 to 200 μg mL^−1^) of either control or YE 500‐elicited extracts. After 48 h incubation, cell viability was evaluated by the MTT assay as previously reported.^18^ The antioxidant activity of the extracts was evaluated by measuring the intracellular reactive oxygen species (ROS) in HaCaT cells treated with 100 μg mL^−1^ of both extract upon induction of oxidative stress, as described by Petruk *et al*.^13^

### Experimental design and statistical analysis

The experimental results of TPC, antioxidant activity (ABTS) and qRT‐PCR analysis were expressed as the mean ± standard error (SE, *n* = 3), whereas the quantitative polyphenol and ursolic acid determination and the disk diffusion, the MTT and DCFDA assays as the mean ± standard deviation (SD, *n* = 3). SD and SE were calculated using Microsoft Excel 2023 software. For the TPC, ABTS and polyphenol and ursolic acid quantitative determination, the YE treatments were evaluated by analysis of variance (ANOVA) using SPSS statistics version 28.0. Tukey's multiple range test was performed to determine the significance of differences between different treatments (*P* < 0.05). For the qRT‐PCR, MTT, DCFDA and disk diffusion assays the YE treatments were evaluated by the ANOVA using GraphPad version 10.0.0. Student's *t*‐test was performed to determine the significance of differences between different treatments (*P* < 0.05).

## RESULTS

### Apple PCC formation and analysis of polyphenol quantity and antioxidant activity

**Figure 1:**
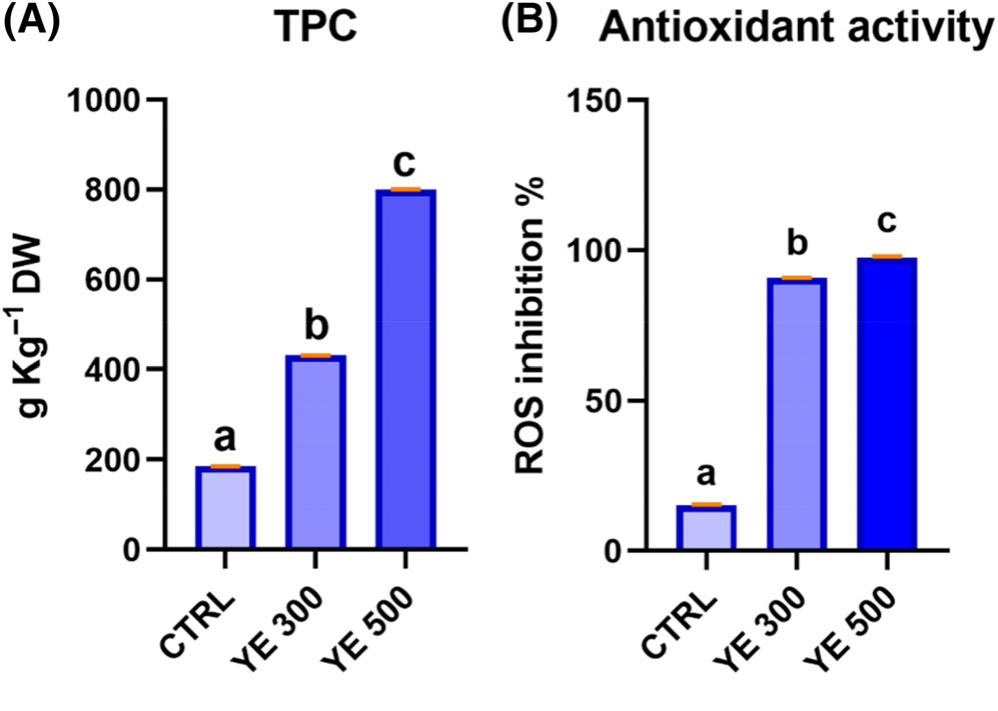
Yeast extract (YE) effect on Annurca peel‐derived calli on (A) total polyphenol content (TPC) and (B) antioxidant activity %. Superscript letters indicate significant differences by Tukey's multiple comparison test (*P*< 0.05). CTRL indicates non‐elicited cultures whereas YE 300 and YE 500 indicate elicited cultures.

Callus induction from the Annurca apple peel determined the production of yellow calli with friable appearance (Supporting Information Fig. S1). After a complete formation of callus cultures, the effect of two concentrations of YE, 300 and 500 mg L^−1^, was investigated. The treatment with YE 500 did not affect the growth of peel‐derived calli, whereas the use of a lower concentration of the elicitor seemed to significantly reduce the biomass growth (Fig. S2). YE treatment also influenced the accumulation of polyphenols in the calli (Fig. 1(A)). Specifically, YE 500 triggered the highest accumulation of total polyphenols, showing an approximately four‐fold increase compared to non‐elicited calli, whereas the use of YE 300 resulted in a two‐fold increase of total polyphenols. Consistent with the TPC, the highest antioxidant activity was observed in PCCs elicited with YE 500 as a seven‐fold increase was observed compared to the non‐elicited calli (Fig. 1(B)).

### Metabolite accumulation in non‐ and YE‐elicited PCCs

Chlorogenic acid and phloridzin levels, proportionally increased with the concentration of YE. Upon YE 500 treatment, a significant increase (49‐fold increase) in chlorogenic acid concentration was observed compared to non‐treated calli (Table 1). At the same YE concentration, phloridzin reached 0.441 mg g^−1^ DW, while in the control the amount of phloridzin was below the calculated LOQ (0.002 mg mL^−1^). YE elicitation also stimulated (−)‐catechin biosynthesis, since this molecule was detected only after YE treatment, while procyanidin B2 accumulation was negatively affected by the elicitation (Table 1). Besides polyphenols, triterpenoids are also recognized to be very abundant in apples.^7^ To evaluate the potential of YE in promoting triterpenoids biosynthesis, the accumulation of ursolic acid was analysed as it is one of the most synthetized in apple.^19^ YE elicitation positively affected ursolic acid content in apple calli and the maximum concentration was achieved in the cultures elicited with YE 300 (two‐fold increase compared to the control).

**Table 1:** Effect on polyphenol content upon yeast extract (YE) elicitation^†^

Compounds	CTRL	YE 300	YE 500
Chlorogenic acid	0.023 ± 0.000^a^	0.545 ± 0.009^b^	1.120 ± 0.030^c^
Phloridzin	LOQ	0.096 ± 0.024^a^	0.441 ± 0.016^b^
Procyanidin B2	0.267 ± 0.004^a^	LOQ	0.167 ± 0.005^b^
(−)‐Catechin	ND	0.143 ± 0.013^a^	0.146 ± 0.001^a^
Ursolic acid	5.790 ± 1.530^a^	13.46 ± 2.080^b^	9.750 ± 2.130^a^

*Note*: CTRL indicates non‐elicited cultures whereas YE 300 and YE 500 indicate elicited cultures. ND and LOQ are molecules not detected or at limit of quantification, respectively.

^†^ Data are expressed as mean value (milligrams of component) g^−1^ dry weight (DW) of callus ± standard deviation (SD). Mean values with different superscript letters are significantly different by Tukey's multiple comparison test (*P* < 0.05) calculated along the lines.

### Expression levels of genes involved in phloridzin and ursolic acid biosynthesis

**Figure 2:**
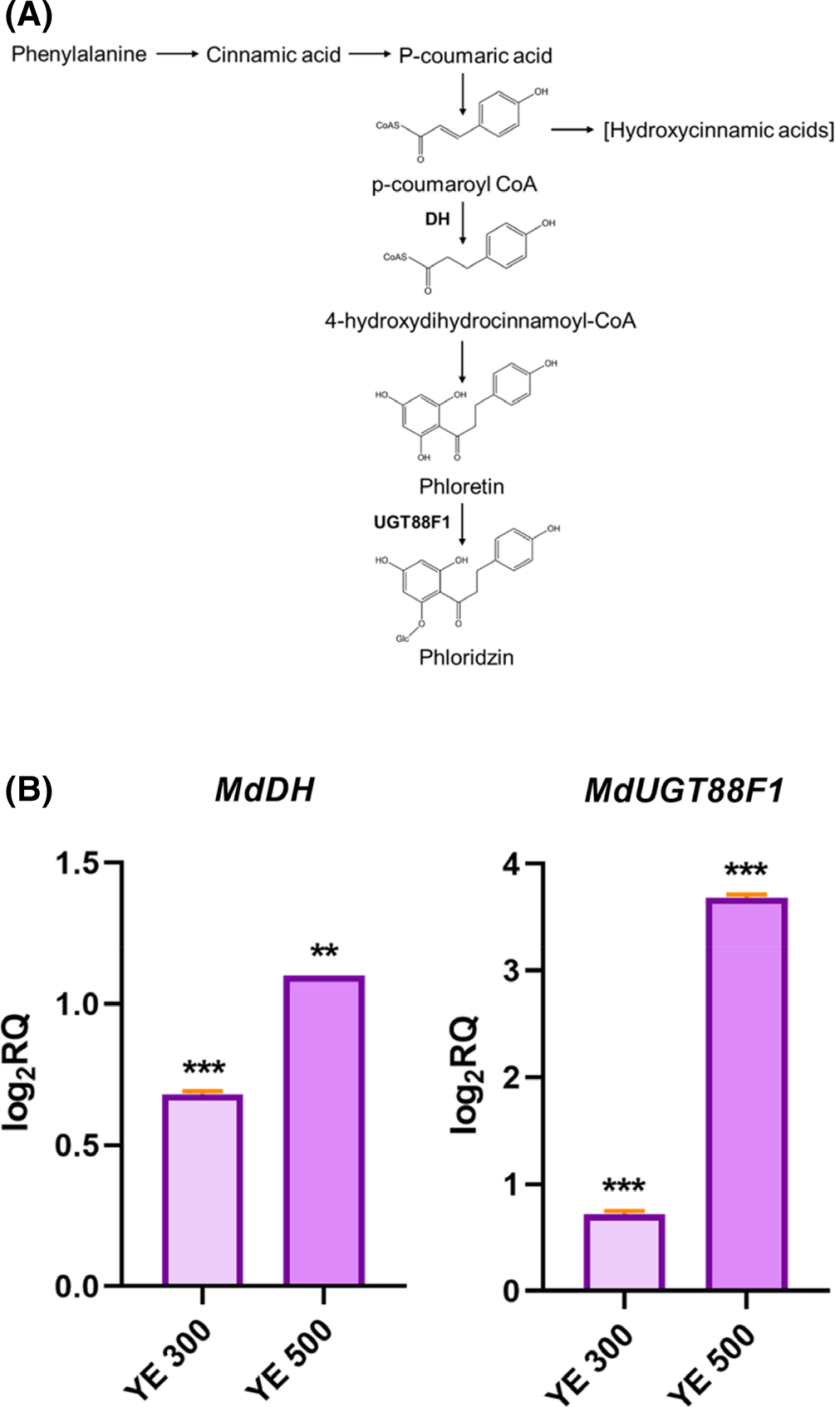
(A) Phloridzin pathway; (B) Yeast extract (YE) effect on the genes *MdDH* and *MdUGT88F1*, related to phloridzin biosynthesis in Annurca peel‐derived calli. The relative quantity of gene expression levels is defined as log_2_RQ. Asterisks denote statistically significant differences of each treatment compared to the control samples that here are not shown (**P < 0.01; ***P < 0.001) by Student *t*‐test. YE 300 and YE 500 indicate the elicited cultures.

**Figure 3:**
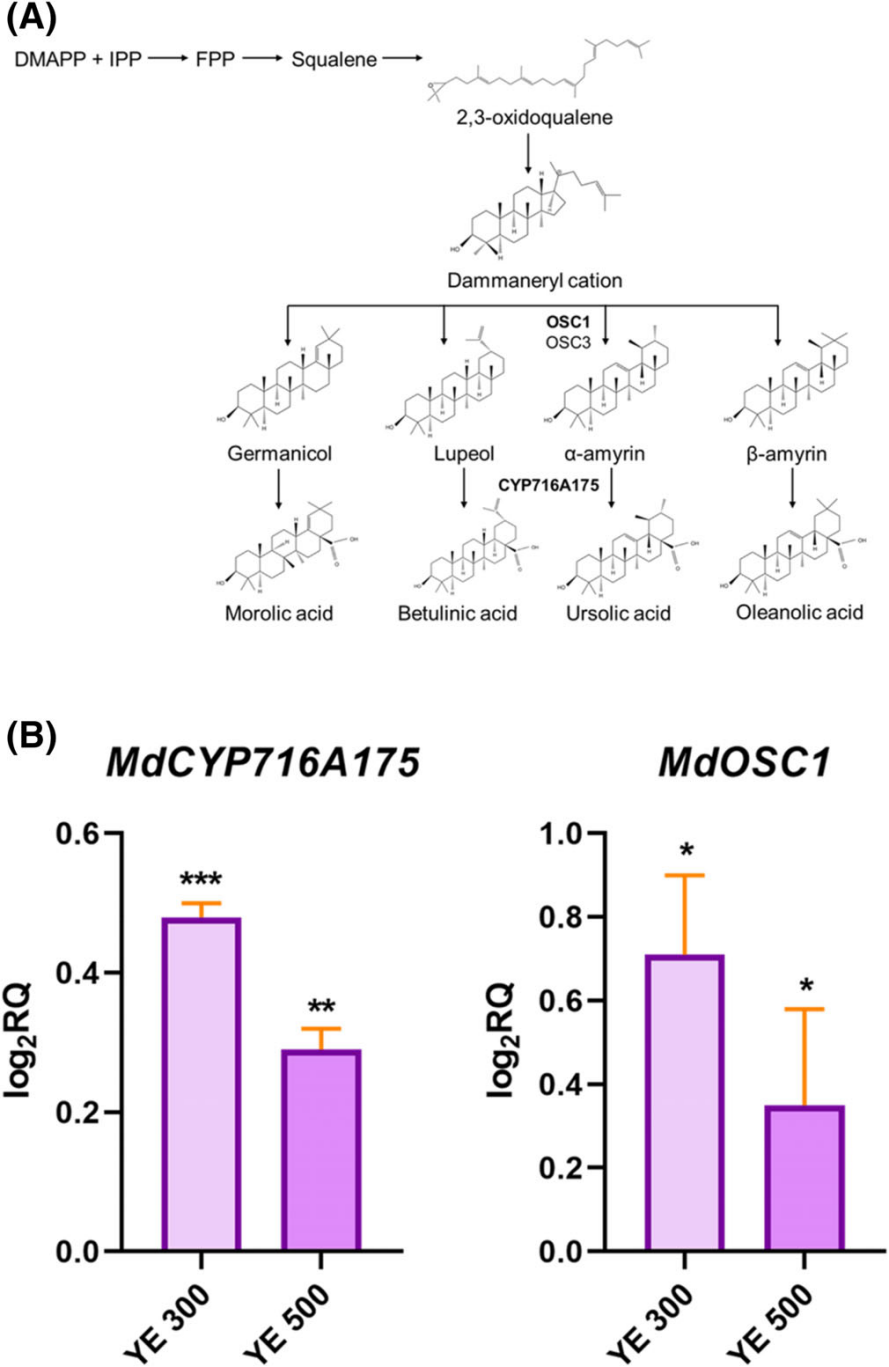
(A) Ursolic acid pathway; (B) Yeast extract (YE) effect on the genes *MdCYP716A175* and *MdOSC1*, related to ursolic acid biosynthesis in Annurca peel‐derived calli. The relative quantity of gene expression levels is defined as log_2_RQ. Asterisks denote statistically significant differences of each treatment compared to the control samples that here are not shown (*P < 0.05; **P < 0.01; ***P < 0.001) by Student *t*‐test. YE 300 and YE 500 indicate the elicited cultures.

Phloridzin and ursolic acid are the most affected compounds by YE elicitation, but also two of the most abundant molecules in apple peel.^20^ In light of this, the expression level of genes encoding key enzymes of the phloridzin and ursolic acid biosynthetic pathway (Figs 2(A) and 3(A)) was investigated. The *MdDH* gene, encoding 2‐alkenal reductase (NADP(^+^)‐dependent)‐like, and *MdUGT88F1* gene, encoding UDP‐glucose: phloretin 2′‐O‐glucosyltransferase, involved in phloridzin biosynthesis, were up‐regulated. In particular, after YE 500 elicitation a four‐fold increase in the expression of *MdUGT88F1* gene was recorded (Fig. 2(B)). The expression of *MdOSC1* gene, encoding the oxydosqualene cyclase 1, and *MdCYP716A175* gene, encoding the triterpene monooxygenase, was analysed to investigate the ursolic acid biosynthetic pathway. The expression of both genes increased after elicitation (Fig. 3(B)). Perfectly in line with the metabolic results, YE 300 produced the most dramatic effect, and a five‐fold gene expression increase for both *MdOSC1* and *MdCYP716A175* was registered compared to the control.

### Assessment of antimicrobial activity of PCC extracts

The antimicrobial potential of Annurca callus extracts was evaluated against foodborne bacteria, both Gram‐positive (*S. aureus* and *E. faecalis*) and Gram‐negative strains (*E. coli*, *S. enterica*, *L. monocytogenes* and *B. cereus*). Bovine serum albumin (BSA) and a mixture of common antibiotics were used as negative and positive controls. Extracts from the non‐elicited calli were found to be effective against all strains. In particular, compared to the antibiotic mixture, the use of these extracts induced a two‐fold increased effect against *E. faecalis*, *E. coli*, *L. monocytogenes*, a three‐fold increased effect against *S. aureus* and *S. enterica*, and a four‐fold increased effect against *B. cereus*. Nevertheless, an enhanced inhibition in bacterial growth was observed using the elicited extract (Table 2).

**Table 2:** Inhibition diameter (mm) of Gram positive and negative strains induced by peel‐derived callus extracts.^†^

Bacterium species	Antibiotic mixture (ampicillin and clavulanic acid)	CTRL	YE 500
*Staphylococcus aureus*	10 ± 0.04	29 ± 0.05*	31 ± 0.02**
*Enterococcus faecalis*	11 ± 0.1	20 ± 0.07*	22 ± 0.06*
*Escherichia coli*	11 ± 0.06	24 ± 0.08*	19 ± 0.02*
*Salmonella enterica*	9 ± 0.3	24 ± 0.05**	25 ± 0.04**
*Listeria monocytogenes*	12 ± 0.07	20 ± 0.1**	22 ± 0.01*
*Bacillus cereus*	9 ± 0.05	35 ± 0.06*	37 ± 0.02**

*Note*: CTRL and YE 500 indicate non‐elicited and elicited callus extract, respectively.

^†^ Data are expressed as mean value ± standard deviation. Asterisks show statistically significant differences of each treatment compared to the control (antibiotic mixture) samples (*P < 0.05; **P < 0.01).

### Biocompatibility and protective effect of peel‐derived callus extracts

**Figure 4:**
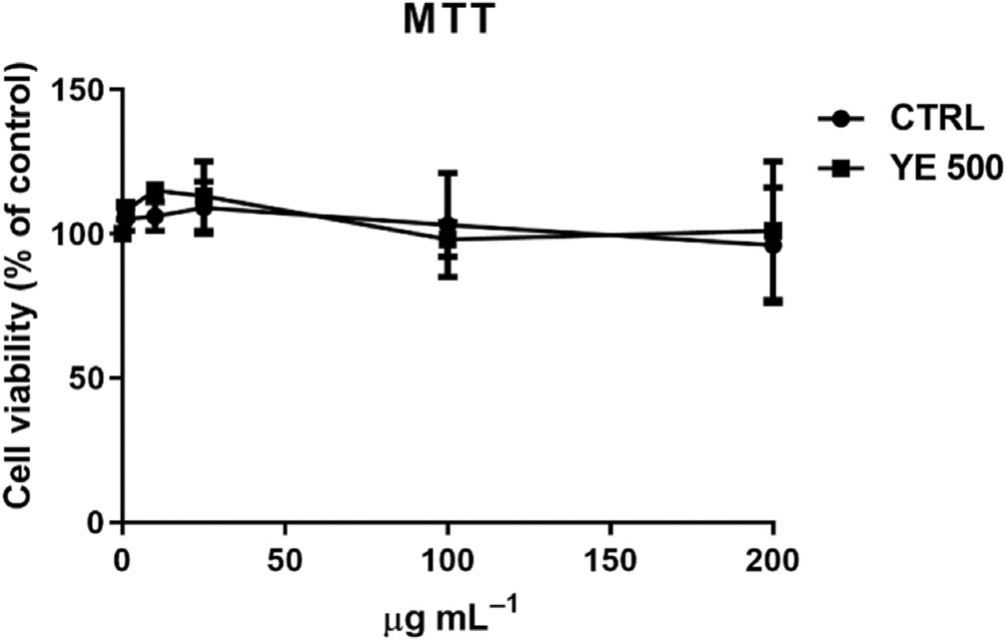
Effect of Annurca peel‐derived callus extracts on cell survival. Dose–response curve of HaCaT cells incubated for 48 h with increasing concentrations (1–200 μg mL^−1^) of control extract (black circles) and YE 500 extract (black squares).

The effect of peel‐derived callus extracts on cells viability was determined. HaCaT cells were incubated with increasing concentration of extracts, and after 48 h cell viability was assessed by the MTT assay. As shown in Fig. 4, no effect on cell viability was observed at any concentration tested, thus confirming that both extracts were fully biocompatible.

**Figure 5:**
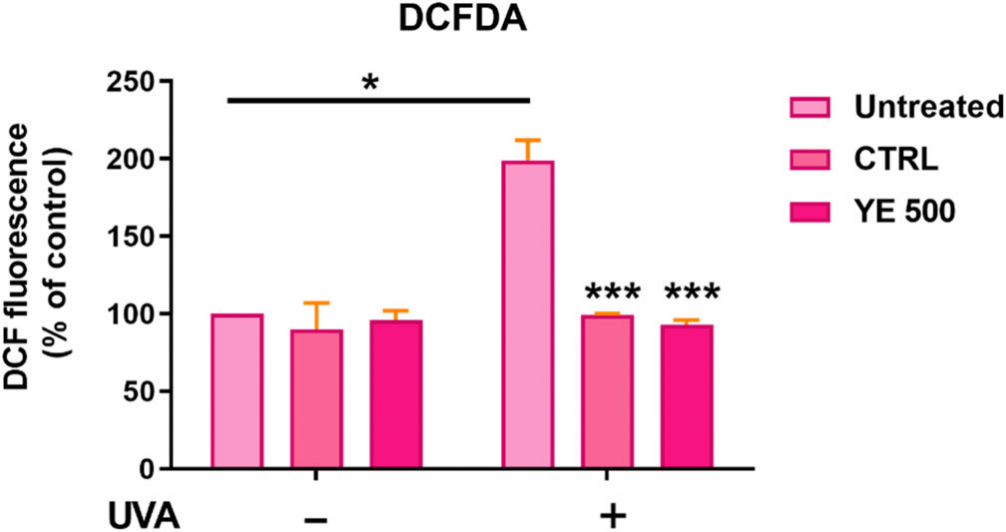
Protective effect of Annurca peel‐derived callus extracts on UV‐A‐stressed HaCaT cells. Light pink bars refer to non‐elicited callus cultures, pink bars refer to cells incubated with control extract, and dark pink bars refer to cells incubated with elicited extract, in the absence (−) or in the presence (+) of UV‐A stress. Values are expressed as percentages compared to non‐elicited callus cultures. Student *t*‐test: *Indicates a statistical difference with respect to untreated cells (*P* < 0.05). ***Indicates a statistical difference with respect to UV‐A‐irradiated untreated cells (*P* < 0.005). Untreated indicates HaCaT cells without the addition of callus extract, whereas CTRL and YE 500 indicate HaCaT cells upon the addition of non‐elicited and elicited callus extract, respectively.

Then, the antioxidant activity was investigated by DCFDA assay.^13^ The incubation with either control or elicited extract, in the absence of oxidative stress, did not alter intracellular ROS levels (Fig. 5). When cells were irradiated with the UV‐A lamp, a significant increase in ROS levels was observed (200%). Interestingly, when cells were pre‐treated with either control or elicited extract, prior UV‐A irradiation, a 100% inhibition in ROS production was observed.

## DISCUSSION

Plant cell cultures offer the advantage of withstanding environmental changes for the continuous production of specific phytochemicals.^21^ Herein, Annurca apple was chosen for PCC production, as it is a prominent edible fruit known for its high accumulation of antioxidant compounds compared to other apple varieties.^22^

In this study, a protocol for inducing callogenesis from the peel of the Annurca apple was developed, obtaining yellow and friable calli. Similarly, callus cultures were previously obtained from Annurca apple pulp,^5^ as well as from other apple varieties such as Golden Delicious and Mela Rosa Marchigiana.^23^ The production of calli with this colouration may be related to the fact that they are usually grown in the dark and accumulate phenolic compounds that prevent the accumulation of darker compounds, including anthocyanins.^24^

One drawback of PCC production is the development of a stable cell line that can consistently guarantee productivity during *in vitro* maintenance. To address this issue, elicitors can be employed to promote secondary metabolism.^25^ Here, YE was selected as elicitor due to its natural stress‐inducing properties and cost‐effectiveness among other available elicitors.^26^ The concentrations used were 300 and 500 mg L^−1^ since these concentrations had been already proved to stimulate the metabolic response in apple‐derived PCCs.^5^ Our results indicate that, in the peel‐derived callus cultures, YE treatment increased the accumulation of TPC and of its related antioxidant activity, but did not significantly alter the callus biomass growth (especially at 500 mg L^−1^), consistently with the effect observed in our previous work.^5^ The peel of the Annurca apple was reported to be enriched in polyphenols, such as chlorogenic acid, (−)‐catechin, (−)‐epicatechin, phloridzin, rutin, quercetin‐3‐glucoside, procyanidin B2, and triterpenic acids such as ursolic, oleanolic, pomolic, betulinic and annurcoic acids.^27^ In the Annurca peel‐derived calli, some of these metabolites, such as chlorogenic acid, phloridzin, procyanidin B2, (−)‐catechin and ursolic acid were identified. The higher level of TPC registered in YE elicited calli was mainly due to the accumulation of chlorogenic acid, as already observed in PCC from Annurca apple pulp. However, in peel‐derived calli, phloridzin accumulation significantly increased upon YE elicitation, while this did not occur in pulp‐derived calli. Moreover, in pulp‐derived calli only YE 500 determined an enhanced biosynthesis of (−)‐catechin and procyanidin B2,^5^ whereas in peel‐derived calli, (−)‐catechin content increased independently from the YE concentration. Moreover, procyanidin B2 content decreased in YE 500‐elicited peel‐derived calli, likely indicating that the YE treatment caused a redirection of the metabolic flux from procyanidin towards phenylpropanoid production. The different metabolic composition of PCCs from the apple pulp and peel might indicate that the two lines maintained the signalling/regulatory mechanism of the matrices.^28^ Indeed, in peel‐derived calli, YE treatment induced an enhanced production of molecules like phloridzin, that usually accumulate in the apple peel after an infection.^29^

The impact of the elicitation was evident on the expression levels of *MdDH* gene encoding the 2‐alkenal reductase, a crucial enzyme for the redirection of the metabolic pathway towards the biosynthesis of dihydrochalcone from the general polyphenol pathway^20^ and *MdUGT88F1* gene, encoding the UDP‐glucose:phloretin 2′‐O‐glucosyltransferase that leads to the glycosylation of phloretin turning it into phloridzin.^30^

The effect of YE treatment on triterpenoid composition was also investigated, analysing the accumulation of ursolic acid, a compound involved in defence mechanisms against biotic stressors.^31^ Here, the highest production of ursolic acid (+132% *versus* control) was obtained after treatment with YE 300. YE treatment also caused a higher expression level of *MdOSC1* genes and *MdCYP716A175* (encoding the oxydosqualene cyclase 1 and the triterpene monooxygenase) which are key genes for ursolic acid production in apple fruit.^32^ The higher levels of phloridzin and ursolic acid recorded in the elicited peel‐derived callus cultures may be linked to the fact that these are specialized bioactive molecules against pathogens,^33^ emphasizing at the same time the efficiency of YE as elicitor.^34^

Accordingly, both ursolic acid and phloridzin were demonstrated to be effective antimicrobial molecules against Gram positive and Gram negative foodborne bacteria.^35^, ^36^ Here, we explored the possibility that bioactive metabolites accumulating in peel‐derived calli may contrast the replication of multi‐drug resistance bacterial strains testing their effects against Gram positive and Gram negative bacterial strains. The extract from non‐elicited calli had already an impressive antibacterial effect, especially against *S. aureus* and *B. cereus*. The effect was ameliorated after YE 500 elicitation, suggesting a possible contribution of phloridzin and ursolic acid to the antimicrobial property of the extract, interacting with the phenolic groups present within the lipid bilayer of bacterium membrane and altering its permeability and integrity.^37^

Overall, Annurca peel extracts have already been shown to reduce bacterial growth^38^ but, to the best of our knowledge, this is the first time that the antimicrobial potential of apple peel callus extract was reported.

Apple peel has also been established to be a source of antioxidant molecules able to counteract UV oxidative stress.^39^ Here, the antioxidant potential of both control and YE 500‐elicited extracts was tested on eukaryotic cells. Our findings revealed a protective effect of both extracts against UV‐A injury; however, no differences in the protective effect were observed between the control extract and the elicited one. Considering our previous work,^5^ one compound that differed between the metabolomic profile of Annurca peel‐ and pulp‐derived calli was procyanidin B2. This compound was not present in pulp‐derived calli and increased with the YE 500‐treatment; conversely, it was already present in peel‐derived calli, and slightly decreased with YE 500‐treatment. This molecule was proved to be able to protect human cells from UV damage, by scavenging oxidative species like ROS and down‐regulating stress‐activated MAPK and apoptosis‐related pathways^40^ but further investigation will better clarify its antioxidant role.

Altogether, our study confirmed the possibility to use calli obtained from the peel of the Italian local variety, Annurca, as a sustainable approach to boost the synthesis of valuable compounds. In particular, the antimicrobial potential of these extracts against foodborne pathogens was demonstrated. The next studies will further investigate the metabolome and the transcriptome of these cell lines after YE treatment to better elucidate the effects of this biotic elicitor on callus cultures.

## CONFLICT OF INTEREST STATEMENT

The authors declare that they have no conflicts of interest.

## Supporting information


**Figure S1.** (A) Annurca peel in the medium for callogenesis induction; (B) peel‐derived calli.
**Figure S2.** YE effect on the biomass growth of Annurca peel‐derived calli.
**Table S1.** Primers for qRT‐PCR.

## Data Availability

The data that support the findings of this study are available from the corresponding author upon reasonable request.
