# Supplementary material for: Elicitor‐mediated simultaneous accumulation of phloridzin and ursolic acid in Annurca apple peel‐derived calli
Source: J Sci Food Agric. 2024 Oct 10;105(3):1783–90. doi: 10.1002/jsfa.13955 (PMC11726598; doi:10.1002/jsfa.13955)
Supplement: Supplementary file 1 — Figure S1. (A) Annurca peel in the medium for callogenesis induction; (B) peel‐derived calli. Figure S2. YE effect on the biomass growth of Annurca peel‐derived calli. Table S1. Primers for qRT‐PCR. [file JSFA-105-1783-s001.docx]

**
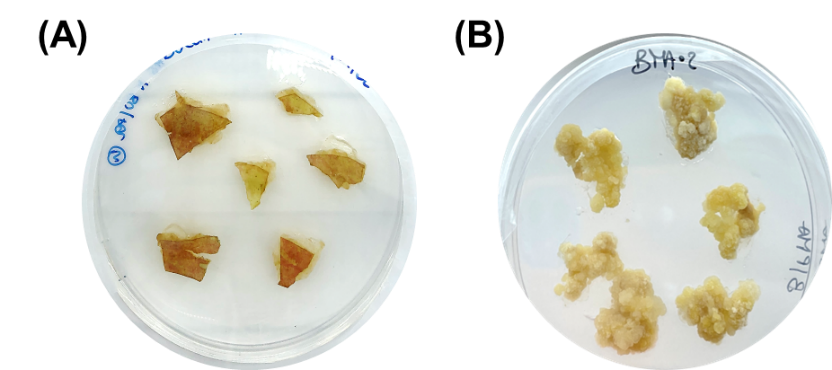
**

**Figure S1.** (A) Annurca peel in the medium for callogenesis induction; (B) peel-derived calli.


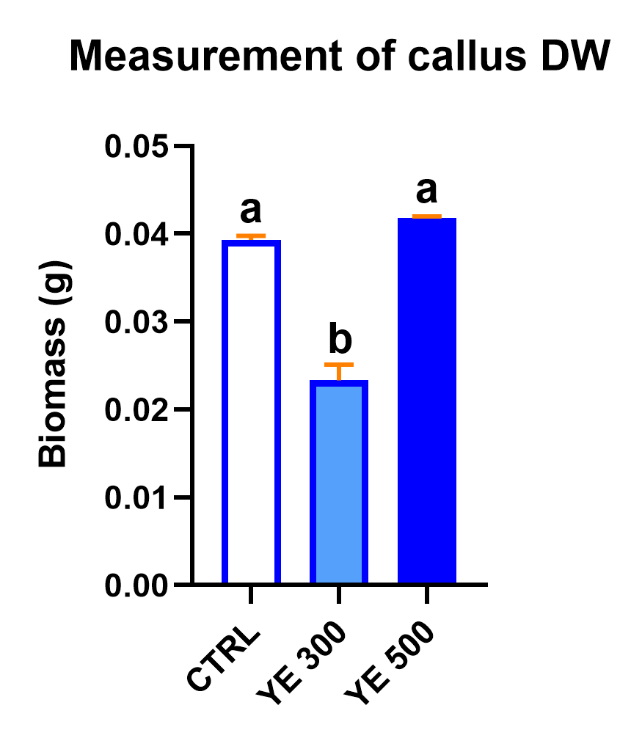


**Figure S2.** YE effect on the biomass growth of Annurca peel-derived calli.

| **Table S1. Primers for qRT-PCR** | | |
| --- | --- | --- |
| **Genes** | **Forward** | **Reverse** |
| MdDH | CGGTCTTCATTTCAGCAGCA | AAGCTGCATCCAAATCAGGC |
| MdUGT88F1 | CTTCTGGTGCCGCAATTCTT | AACGACCCTGCTTATTCGGA |
| MdOSC1 | CCGTGGTGGTGCGTACTATT | GATGATGCTTGCTTGCTGGG |
| MdCYP175A716 | GGGCTGGGAAAACAAGAAGG | TCGATATGATTCCCGAGGCC |
